# Epoxy Coatings Containing Modified Graphene for Electromagnetic Shielding

**DOI:** 10.3390/polym14122508

**Published:** 2022-06-20

**Authors:** Marius Gabriel Bontaș, Aurel Diacon, Ioan Călinescu, Mădălina Ioana Necolau, Adrian Dinescu, Gabriela Toader, Raluca Ginghină, Alexandru-Mădălin Vizitiu, Valentin Velicu, Petru Palade, Marcel Istrate, Edina Rusen

**Affiliations:** 1 Faculty of Chemical Engineering and Biotechnologies, University Politehnica Bucharest, Gh. Polizu Street, 011061 Bucharest, Romania; bontasmariusgabriel@yahoo.com (M.G.B.); aurel.diacon@upb.ro (A.D.); ioan.calinescu@gmail.com (I.C.); madalinanecolau@gmail.com (M.I.N.); 2 S.C. Protect Chemical S.R.L., 6 Cercetătorilor Street, 042024 Bucharest, Romania; 3 Advanced Polymer Materials Group, University Politehnica of Bucharest, Gh. Polizu Street, 011061 Bucharest, Romania; 4 National Institute for Research and Development in Microtechnologies (IMT-Bucharest), 126A Erou Iancu Nicolae Street, 023573 Bucharest, Romania; adrian.dinescu@imt.ro; 5 Military Technical Academy “Ferdinand I”, 39-49 George Coșbuc Boulevard, 050141 Bucharest, Romania; nitagabriela.t@gmail.com; 6 Research and Innovation Center for CBRN Defense and Ecology, 225 Soseaua Oltenitei, 041327 Bucharest, Romania; ginghinaraluca@gmail.com; 7 Faculty of Electronics, Telecommunications and Information Technology, University Politehnica of Bucharest, 313 Splaiul Independentei, 060042 Bucharest, Romania; vizitiuamadalin@gmail.com; 8 The Special Telecommunications Service, 323A Splaiul Independenţei, 060044 Bucharest, Romania; valentin.velicu91@gmail.com; 9 Faculty of Electrical Engineering, University Politehnica of Bucharest, 313 Splaiul Independentei, 060042 Bucharest, Romania; 10 National Institute of Materials Physics, P.O. Box MG-7, 077125 Bucharest, Romania; palade@infim.ro; 11 S.C. Stimpex S.A., 46-48 Nicolae Teclu Street, 032368 Bucharest, Romania; office@stimpex.ro

**Keywords:** radar absorbing materials, flexible epoxy textile coating, graphene functionalization

## Abstract

This study presents the functionalization and characterization of graphene and electromagnetic interference (EMI) attenuation capacity in epoxy-nanocomposites. The modification of graphene involved both small molecules and polymers for compatibilization with epoxy resin components to provide EMI shielding. The TGA and RAMAN analyses confirmed the synthesis of graphene with a different layer thickness of the graphene sheets. Graphene samples with different layer thicknesses (monolayer, few layers, and multilayer) were selected and further employed for epoxy coating formulation. The obtained nanocomposites were characterized in terms of EMI shielding effectiveness, SEM, micro-CT, magnetic properties, and stress-strain resistance. The EMI shielding effectiveness results indicated that the unmodified graphene and hexamethylene diamine (HMDA) modified graphene displayed the best EMI shielding properties at 11 GHz. However, the epoxy nanocomposites based on HMDA modified graphene displayed better flexibility with an identical EMI shielding effectiveness compared to the unmodified graphene despite the formation of aggregates. The improved flexibility of the epoxy nanocomposites and EMI shielding characteristics of HMDA functionalized graphene offers a practical solution for textile coatings with microwave absorbing (MA) capacity.

## 1. Introduction

The design and development of innovative lightweight and flexible broadband microwave absorbing (MA) materials is one of the current challenges for the academic and industry sectors due to the increased use of electromagnetic waves (EM) in radar systems, civil, and military applications [1,2,3,4]. Microwaves are part of the electromagnetic spectrum with wavelengths from one millimeter to a meter, in the frequency range of 10^9^–10^12^ Hz and they have two components: electric and magnetic [5].

MA materials can be classified into the following two categories: magnetic and dielectric absorbing materials [6,7]. A perfect MA material should be lightweight, thin, and able to cover a broad frequency range. MA materials have excellent absorption capacities, but the densities are usually too high. In contrast, usually dielectric absorbers are much lighter in weight, but they do not possess the absorptivity capacity of magnetic absorbers. Because the absorption loss is a function of conductivity [8,9], dielectric permittivity [10,11,12], and magnetic permeability of the material [13], the absorption loss in the material is caused by the heat loss attributed to the alignment of electric and/or magnetic dipoles in the EM field. A key challenge is the ability to tune the interaction between microwaves and matter by electrical and magnetic means [14].

Polymer-based composites could meet all these requirements [15,16,17]. For military applications, MA materials can be used in various forms such as paints [18] and composites for reducing the radar cross-section [19] of various strategic targets such as aircraft, missiles, etc. [20]. Many polymer-based composites [21,22] have been recently developed to obtain materials and understand their EMI shielding behavior [23,24].

In recent years, graphene has attracted lots of attention for its potential application in MA; the attractive potential properties of graphene for MA performance are probably attributed to the following reasons: the ultrathin thickness and optical transmittance property of graphene can not only decrease the density of the MA materials but also weaken the skin effect effectively; the resonance effect resulted from the layered and high porous morphology as well as the high specific surface area could be very beneficial to the absorption and attenuation of the microwave; the interfacial polarization caused by the large interface can also be helpful for the absorption of the microwave [25,26,27,28,29].

Epoxy resins are the most important thermosets, widely used as adhesives, coatings, and as a polymer matrix to obtain polymer nanocomposites [20,30,31,32]. It also has good mechanical properties, such as high compression strength, and high durability in terms of fatigue and corrosion resistance. Its permeability to liquids is generally very low, and its curing times are fast. One of the most common materials is called iron ball paint, which contains tiny metal-coated spheres suspended in epoxy-based paint [33]. The spheres are coated with ferrite or carbonyl iron. When EM enters iron ball paint it is absorbed by the ferrite or carbonyl iron molecules, which causes them to oscillate. The molecular oscillations then decay with the release of heat, and this is an effective mechanism of damping electromagnetic waves. The tiny amount of heat generated by the oscillations is conducted into the fuselage where it dissipates.

Our study deals with the synthesis of new EMI shielding materials based on epoxy polymers and chemically modified graphene. The graphene has been functionalized for better dispersion in the epoxy resin (polymer matrix). In our case, the functionalization takes place with small molecules, respectively with polymers. Samples of graphene with different layer thicknesses (monolayer, few layers, and multilayer) were selected and further employed for epoxy coating formulation. Thus, the EMI shielding attenuation capacity of the nanocomposites containing modified graphene was evaluated. Additionally, the mechanical properties of the epoxy composites were assessed given the final objective to obtain an efficient EMI shielding solution capable to be used in textile coatings. Thus, modified graphene-based materials were demonstrated to confer both EMI shielding effectiveness and suitable mechanical properties, making them one of the most appropriate materials for improving MA capacity in the future.

## 2. Materials and Methods

### 2.1. Materials

Graphene nanoplatelets (5 μm wide, average 5 nm thickness, bulk density of 0.03–0.1 g/cc, carbon content > 99.5 wt%, oxygen content < 1%, and residual acid content < 0.5 wt%) (G) (Emfutur Technologies Ltd., Castello, Spain) were used as received. Nitric acid (68%, Sigma-Aldrich, St. Louis, MO, USA), sulfuric acid (95–98%, Sigma-Aldrich), glycidyl methacrylate (GMA, Sigma-Aldrich), N,N’-dimethylformamide (DMF, Sigma-Aldrich), lauryl peroxide (LP, Sigma-Aldrich), acetone (Sigma-Aldrich), methanol (Sigma-Aldrich), hexamethylene diamine (HMDA, Sigma-Aldrich), and acrylamide (Sigma-Aldrich) were used as received. Methyl methacrylate (MMA, Sigma-Aldrich) was purified through vacuum distillation. Hexyl methacrylate (HexylMA, Sigma-Aldrich) was purified by passing through an alumina column. Benzoyl peroxide (BP, Sigma-Aldrich) was recrystallized by dissolution in CHCl_3_ and precipitation in methanol.

### 2.2. Methods

**(a)**  **Functionalization of Graphene with small molecules**

**Graphene oxide** was prepared according to a modified method. Briefly, 1 g of graphene was dispersed in 30 mL of H_2_SO_4_ (95–98%), then 10 mL of HNO_3_ (68%) was slowly added and the reaction mixture was stirred for an additional 2 h. After, 100 mL H_2_O was added dropwise, and the reaction mixture was let to cool down under stirring before the addition of another 200 mL H_2_O. The reaction mixture was then neutralized using NaOH until pH 7 was achieved and the obtained GO was separated by filtration and washed thoroughly with water.

**Synthesis of Graphene-G-BP.** In a round bottom flask, 1 g of graphene was dispersed in 60 mL of DMF using an ultrasonic processor (Hielscher, Teltow, Germany, UP50H) followed by the addition of 0.5 g benzoyl peroxide. The reaction mixture was heated to 80 °C under nitrogen and kept under stirring for 4 h. The product was separated by vacuum filtration and washed thoroughly with methanol and acetone.

**Synthesis of G-HMDA.** In a round bottom flask, 1 g of graphene was dispersed in 60 mL of DMF using an ultrasonic processor (Hielscher, UP50H) followed by the addition of 10 g HMDA. The reaction mixture was heated to 80 °C under nitrogen and kept under stirring for 4 h. The product was separated by vacuum filtration and washed thoroughly with water, methanol, and acetone before being dried.

**(b)**  **Functionalization of Graphene with polymer chains**

**Synthesis of G-PAM.** In a round bottom flask, 1 g of graphene was dispersed in 60 mL of DMF using an ultrasonic processor (Hielscher, UP50H) followed by the addition of 10 g acrylamide and 0.1 g of LP. The reaction mixture was heated to 80 °C under nitrogen and kept under stirring for 4 h. The product was separated by vacuum filtration and washed thoroughly with DMF, water, and acetone before being dried.

**Synthesis of G-PGMA.** In a round bottom flask, 1 g of graphene was dispersed in 60 mL of DMF using an ultrasonic processor (Hielscher, UP50H) followed by the addition of 10 mL GMA and 0.1 g of LP. The reaction mixture was heated to 80 °C under nitrogen and kept under stirring for 4 h. The product was separated by vacuum filtration and washed thoroughly with DMF, water, and acetone before being dried.

**Synthesis of G-PHexylMA.** In a round bottom flask, 1 g of graphene was dispersed in 60 mL of DMF using an ultrasonic processor (Hielscher, UP50H) followed by the addition of 10 mL hexylMA and 0.1 g of LP. The reaction mixture was heated to 80 °C under nitrogen and kept under stirring for 4 h. The product was separated by vacuum filtration and washed thoroughly with DMF, water, and acetone before being dried.

**(c)**  **Epoxy coating formulation**

The epoxy coating formulation involved the use of **EPOTEC YD 011X75** (Epoxy Technology, Billerica, MA, USA) (a bisphenol-A based epoxy resin), **dioctyl adipate** (BASF) (as plasticizer), **EPAMINE PC13** (PO.INT.ER) (as hardener), **BP-183-B** (Zhejiang Huate Group, Huzhou, China) (organic bentonite as rheology modifier), and **IOX B-03** (LANXESS) (synthetic iron oxide-Fe_3_O_4_). The resin: hardener ratio was set at 5:1; a 1:1 toluene: acetone solvent mixture was used to facilitate the dispersion of the graphene and modified graphene either in the resin (**G, GO, G-PGMA**) or in the hardener component (**G-HDMA**) and other fillers (**BP-183-B** and **IOX B-03**). The concentration of the EMI active fillers is presented in Error! Reference source not found. The coating was applied on polyester woven material (used for EMI shielding efficiency) and on polyethylene sheets from which the epoxy films were recovered and used for the characterization of the mechanical properties. The deposition was performed by doctor blade technique to ensure a reproducible film thickness.

### 2.3. Characterization Methods

The polymer nanocomposite films were investigated by optical microscopy using an Olympus BX41 light microscope (Olympus Corporation, Tokyo, Japan) equipped with live view E330 7.5 MP Digital SLR Camera and QuickPhoto Micro 2.3 software (PROMICRA, Prague, Czech Republic). The images were collected in transmission mode.

The morphology of the nanocomposite films was also investigated by SEM (scanning electron microscopy) using a Tescan Vega II LMU SEM instrument (TESCAN, Brno, Czech Republic) at 10 keV acceleration voltage.

The distribution of the graphene and iron oxide particles inside the epoxy matrix was examined via micro-CT technique. The SkyScan micro-CT attachment allowed for converting the Tescan Vega II LMU SEM to an X-ray microtomograph for non-destructive imaging and for measuring of the object’s internal microstructure of specimens. Analysis parameters: Exposure time—4 s per projection at electron beam currents of 100 nA; accelerating voltage—30 KeV; step size −1°; scanning time—24 min. Reconstruction was performed by the NRecon program, which used float-point data values for internal calculations during reconstruction and afterward allowed the operator to define the density window as a range of the reconstructed values. The full set of reconstruction results was visualized by the program DataViewer^®^ 2D/3D Micro-CT Slice Visualization (Micro Photonics Inc., Allentown, PA, USA).

Raman spectra of the functionalized graphene were recorded on a DXR Raman Microscope (Thermo Scientific, Waltham, MA, USA) by 473 nm laser line.

The thermogravimetric analyses (TGA) were performed using Netzsch TG 209 F3 Tarsus (Erich NETZSCH GmbH & Co. Holding KG, Selb, Germany) equipment considering the next parameters: nitrogen atmosphere flow rate, 20 mL min^−1^; samples mass, ~3 mg; temperature range, room temperature −900 °C; and heating rate, 10 °C min^−1^ in an alumina crucible.

Stress-strain curves were obtained using an Instron 3382 testing machine (Instron, Norwood, MA, USA). The samples were prepared for the tensile tests by cutting the nanocomposite films (without woven polyester). The tests were carried out according to the international standard ASTM D3039/D3039M-08. For each specimen, the rate of the extension was set at 500 mm/min, and the separation of the initial jaws was set at 50 mm (plain jaw faces). For each type of nanocomposite film, five tensile tests were carried out and the average of the measured values and the standard deviation for each point was registered. A comparative multigraph containing all the true stress/true strain values characteristic for each synthesized material was plotted to evaluate the influence of the nanofiller on their mechanical properties. This multigraph was designed to show only the curves with the closest parameters to the mean values from each set of specimens. 

Hysteresis loops at 300 K were measured using a SQUID magnetometer (Magnetic Properties Measurement System, QD-MPMS-XL-7AC, manufactured by Quantum Design, San Diego, CA, USA) running under the RSO (Reciprocating Sample Option) mode. This apparatus allows performing measurements with a magnetic moment resolution up to 10^−7^ emu. For magnetic measurements, the epoxy thin film strips used were closed in small sample holders.

**Figure 1:**
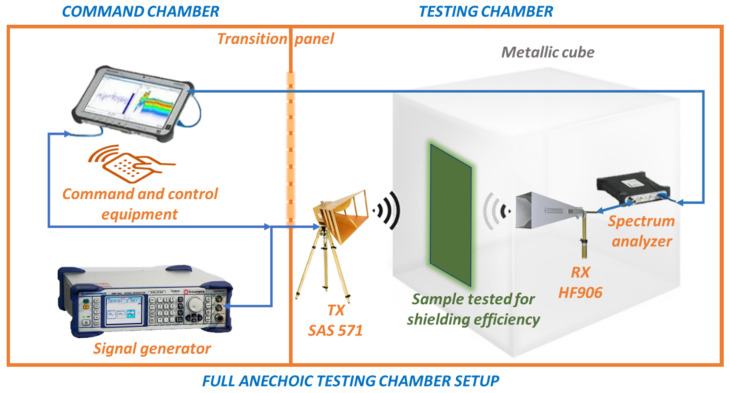
Experimental setup for assessing shielding efficiency of the samples.

The ability of the synthesized materials to attenuate radar waves in the domain of 8–12 GHz was investigated as follows: A full-anechoic chamber (Figure 1), consisting of two distinct areas (command chamber and testing chamber) separated by a transition panel was utilized for performing the measurements. A Rohde & Schwarz SMB100A generator and a command equipment were placed in the command chamber. While in the testing chamber, we placed a metallic cube provided with a slot (A4 dimensions) in which the tested material was fixed. The transfer of the emitted signal was achieved through the transition panel situated between the two above mentioned chambers. Inside the metallic cube, we placed a Tektronix spectrum analyzer (RSA range) connected to a HF906 horn antenna for the reception of the signal to evaluate the shielding efficiency of the tested material. For the emission of the signal, in the testing chamber we also positioned a Horn SAS 571 antenna, which was placed outside the cube, in front of the slot where the tested material was fixed. The antennas have been chosen so that their working ranges cover the range of interest for these materials. The transfer of information from the spectrum analyzer to its control equipment located in the control chamber was done utilizing an optic fiber, the transition being made through a waveguide located between the two chambers. Given that in radar applications the range of interest is in the domain of 8 ÷ 12 GHz, the materials were tested in the range 7 ÷ 12.75 GHz by utilizing the above-described setup, schematically illustrated in Figure 1. To assess the shielding effect of the tested material fixed in the slot of the metallic cube, two measurements were performed: a reference measurement (when the slot provided for fixing the samples was empty, allowing the signal to pass untainted) and the measurement of the sample (when the slot provided was covered by the material subjected to this test and the signal passed through it).

The EMI shielding efficiency (*SE*) is the result of three mechanisms, reflection loss, absorption loss, and multiple reflections loss.

(1) $$SE_{total}=SE_{Refl}+SE_{Abs}+SE_{multiple\;refl}$$

To calculate the *SE_total_*, Equation (2) was used where *E_i_* and *E_t_* are the incident and transmitted electric fields, respectively.

(2) $$SE_{total}=10\log\left(\frac{P_{i}}{P_{t}}\right)=20log\left(\frac{E_{i}}{E_{t}}\right)$$

## 3. Results

The most utilized method to modify G involves obtaining GO, which was then mixed with epoxy resin and deposited on the textile material. This first stage can be used to highlight the physical compatibility between the textile and the modified epoxy resin. Thus, the textile material prior to and after the application of the modified epoxy resin coating was subjected to optical microscopy analysis.

**Figure 2:**
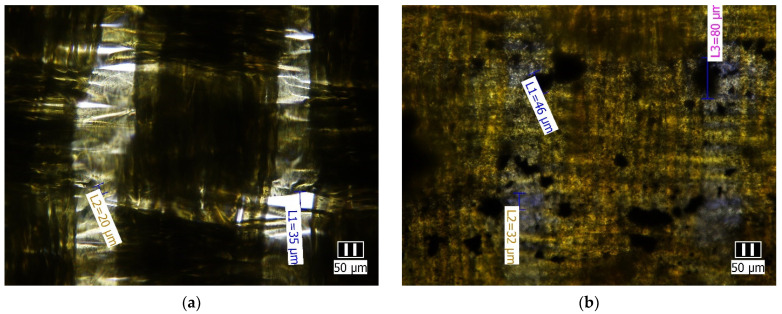
Optical microscopy images: (**a**) unmodified textile material; (**b**) GO-modified epoxy resin.

From the comparative analysis of the images in Figure 2, it can be noticed that the resin coating adhered to almost the entire textile surface and filled the pores in the textile material. However, aggregates of different sizes can be observed, which indicates an improper dispersion of the GO in the epoxy resin matrix. The aggregates can be the result of the rapid reaction between the functional groups of GO and the amine component of the epoxy resin, leading to a reduction in the homogeneity of the deposited coating.

SEM analysis was performed on the two samples for a more precise analysis.

**Figure 3:**
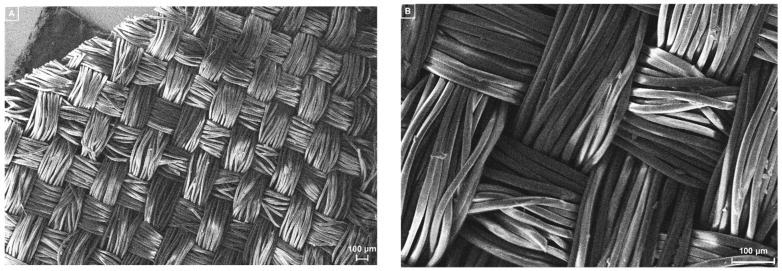

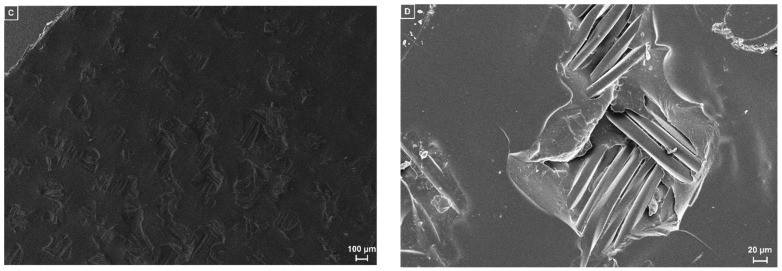
SEM images of unmodified textile material (**A**,**B**) and GO epoxy resin-coated textile material (**C**,**D**).

From the images in Figure 2 and Figure 3 good compatibility between the textile material and the epoxy resin can be observed, however, there are some areas with a non-uniform coating which can be explained by the deposition process.

Addressing the filler capacity to disperse in the resin matrix is a viable solution to further improve the coating quality. For this reason, several methods for graphene functionalization by the introduction of functional groups or polymer grafting were explored. The efficient dispersion and filler compatibility with the epoxy resin are critical aspects that affect the mechanical and microwave absorbing characteristics of the coating. The introduction of functional groups involved the oxidation of graphene, grafting of radical species obtained by the degradation of benzoyl peroxide, and the addition of hexamethylenediamine units to the graphene backbone. The polymer grafting assumed the polymerization in the presence of graphene. The selected monomers were methyl methacrylate (PMMA), glycidyl methacrylate (PGMA), hexyl methacrylate (PhexylMA), and acrylamide (PAM).

**Figure 4:**
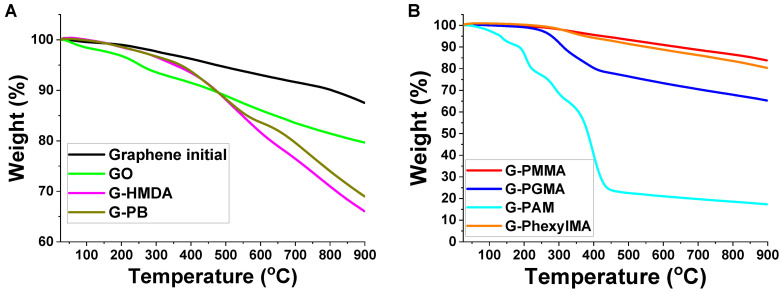
TGA analyses of the functionalized graphene (**A**) small molecules/functional groups and (**B**) polymers.

Thermogravimetric analyses (TGA) were performed to confirm the functionalization of the graphene (Figure 4).

In Figure 4 are presented the comparative TGA analyses for the modified graphene with small molecules/functional groups (A) and with polymers (B). Both types of functionalization aim for the facilitation of graphene dispersion in the epoxy resin, and they can permit a chemical interaction. The analysis of the spectra reveals that the highest thermal resistance was registered for the initial graphene. Considering the first modification approach, small molecules or functional groups, the introduction of new functionality on the graphene backbone results in a decrease of thermal stability. Both HMDA and PB afforded similar results with a residual mass of over 65% at 900 °C. As expected, the oxidation of graphene resulted in the introduction of functional groups hydroxyl [34,35], carboxyl [34,36,37], and oxirane [38], but the registered weight loss is lower than that afforded by the graphene functionalized by the radical grafting of benzoyl peroxide radicals and HMDA addition. The TGA results for polymer functionalized graphene indicate a relatively low polymer to graphene ratio in the case of methyl methacrylate and hexyl methacrylate. Nevertheless, in the case of glycidyl methacrylate and acrylamide this ratio increases. This observation can be explained by the difference in reactivity between the monomers and the competition between the radical trapping action of graphene and the homopolymerization process of the monomer.

**Figure 5:**
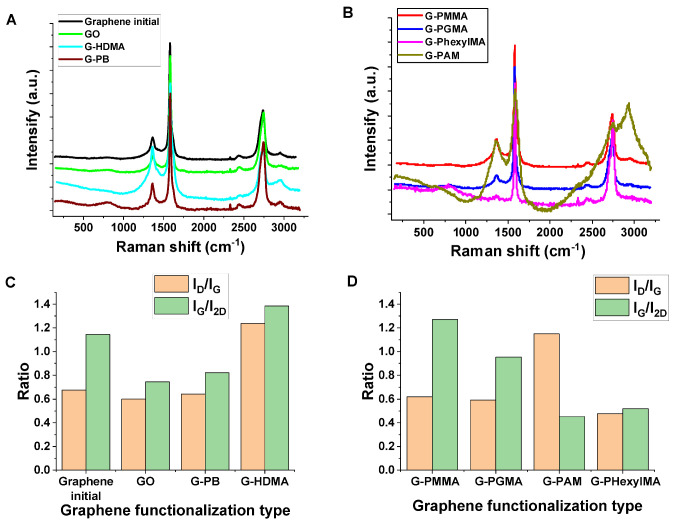
RAMAN spectra, I_D_/I_G_, and I_G_/I_2D_ ratio for the functionalized graphene: initial graphene and functional groups modified graphene (**A**,**C**), polymer functionalized graphene (**B**,**D**).

RAMAN spectroscopy was performed on the modified graphene to confirm the chemical modification of the graphene (Figure 5).

RAMAN spectra analysis of graphene can give valuable information regarding the degree of functionalization, or defect introduction, through the comparison of the intensity of the D (1360 cm^−1^) and G (1575 cm^−1^) bands. Thus, based on the I_D_/I_G_ ratio (Figure 5C,D) we can notice that the highest degree of functionalization was obtained in the case of HDMA and PAM. This observation coincides with the TGA results, confirming the covalent grafting of the small molecule and polymer units.

**Scheme 1:**
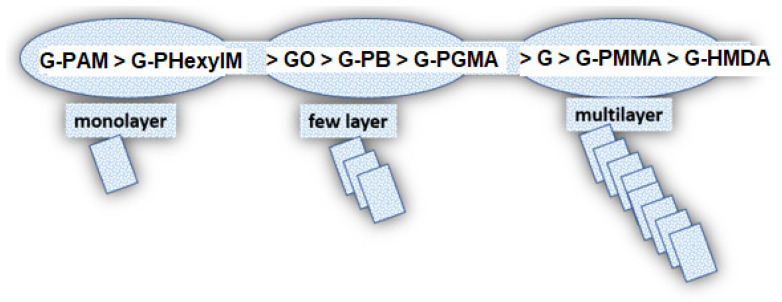
Graphical representation of the graphene stacks’ thickness depending on the functionalization technique.

RAMAN spectra also offer information on the thickness of the graphene sheets and their variation induced by the functionalization technique can be ascertained using the ratio between the G and 2D (2680 cm^−1^) bands. Using the I_G_/I_2D_ ratio (Figure 5C,D) it can be noticed that in the case of PAM a high degree of functionalization also leads to the obtaining of thinner graphene structures. Thus, during the polymerization process the exfoliation of the graphene layers also takes place towards a monolayer structure (the I_G_/I_2D_ value being <0.5). Similarly, for the G-PHexylMA derivative, a similar process is observed, despite the lower functionalization degree. This can be explained by the steric effect of the hexyl unit. In contrast, in G-PGMA and G-PMMA a higher thickness of the graphene stacks was observed. Thus, although G-PMMA and G-PHexylMA display a similar functionalization degree, the thickness of the graphene sheets is different. Another explanation for this aspect resides in the competition during the functionalization process between the radical trapping by the graphene and the homopolymerization of the monomer units. Thus, MMA is more susceptible to homopolymerization compared to hexyl methacrylate and the radical species are more stable than in the case of acrylamide. Surprisingly, in the case of the HDMA, although a high degree of functionalization was obtained, from the I_G_/I_2D_ ratio a multilayer graphene structure was observed. This can be explained by the bifunctionality of the HMDA, which can be intercalated between the graphene sheets and react with both layers. A graphical representation of the obtained structures is presented in Scheme 1.

**Figure 6:**
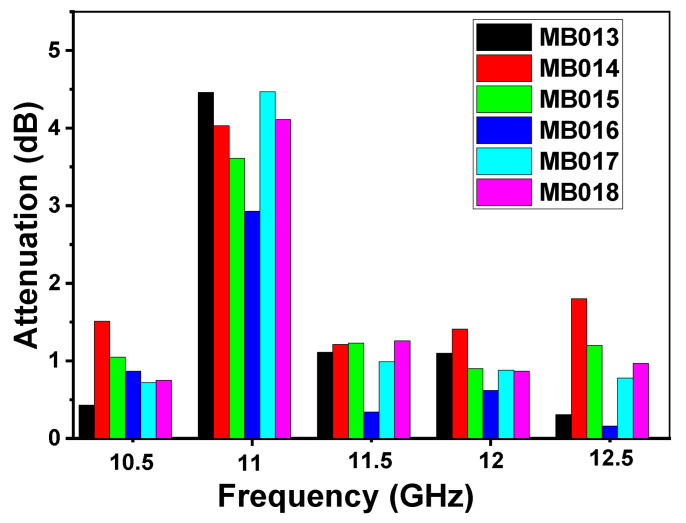
Electromagnetic shielding attenuation dependence on the frequency.

The practical aim of this study consisted in the synthesis, characterization, and utilization of nanocomposite materials with the capacity for EMI shielding. Therefore, four representative specimens G, GO, G-HDMA, and G-PGMA were selected from both the “few layers” and the “multilayer” examples. The selected specimens present the capacity to react with one of the epoxy resin components, while G was selected as the reference point. Iron oxide particles were also added in the nanocomposites to enhance their EMI shielding effectiveness properties. The samples coding is presented in Table 1 while the electromagnetic shielding efficiency is presented in Figure 6.

The EMI shielding results present the highest values at 11 GHz and the order for shielding effectiveness is: MB017 ≈ MB013 > MB018 ≈ MB014 > MB015 > MB016.

Table 2 compares the EMI shielding properties of different composite materials reported in the literature taking into account the matrix, filler and its content, thickness of the sample, and microwave frequency range. Although the values obtained in our case are small, the advantage of a lightweight, cheap solution with good mechanical properties with the capacity of facile scale up can make the materials viable for commercial applications.

The EMI shielding effectiveness depends on the electrical conductivity, permeability, and thickness of the materials [46]. Graphene is an attractive candidate because of its high electrical conductivity, low density, high specific surface area, large aspect ratios, excellent chemical and environmental stability, and mechanical flexibility.

**Figure 7:**
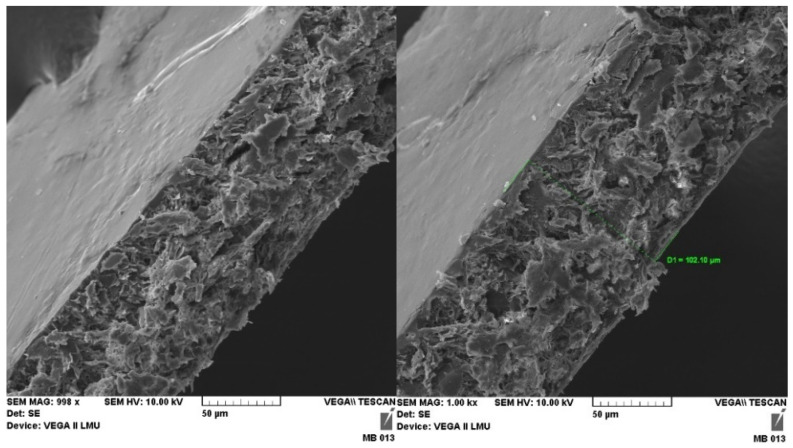
Transversal SEM images of the samples highlighting similar thickness.

The deposition technique permitted the obtaining of coatings with similar thicknesses of around 100 µm (Figure 7), but the difference between the samples consists in the permeability and the electrical conductivity.

**Figure 8:**
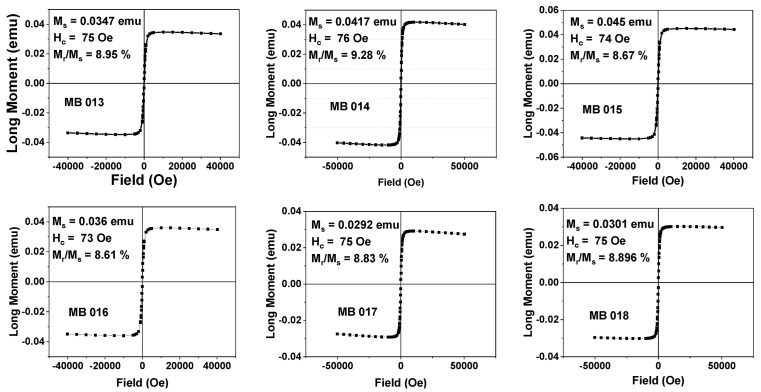
Hysteresis loops measured at 300 K for samples MB013-MB017.

An electromagnetic wave (such as light) has both an electric and a magnetic component. Magnetism is defined as the physical phenomena produced by the moving electric charge. A magnetic field can also induce the movement of the charged particles producing an electrical current, which is the proof of the dependence between magnetism and electric conductivity. The magnetic properties of the samples were assessed to explain their electromagnetic shielding capacity (Figure 8). The values of the coercive field, Hc, and the remanence (the ratio between the remanence magnetization, Mr, and the one of saturation Ms, namely Mr/Ms) are very close for all samples, but the small differences can be correlated as follows: MB014 > MB013 > MB017 > MB018 > MB015 > MB016.

**Figure 9:**
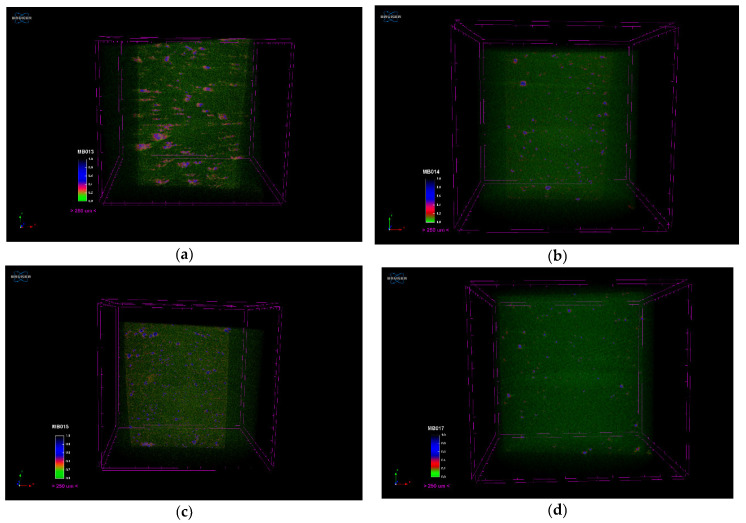
Micro-CT analyses of samples (**a**) MB013, (**b**) MB014, (**c**) MB015, and (**d**) MB017.

It can be easily noticed that the EMI shielding attenuation efficiency decrease does not follow the same trend as the magnetic properties. The last two samples, **MB015** and **MB016**, respect the order for both magnetism and EMI attenuation. These low values could be explained by a higher amount of doping agent which leads to the formation of larger aggregates in the case of **MB015**, therefore larger “multilayer” structures by reaction of the amine group of HMDA with the oxirane of the epoxy component. In the case of **MB016**, although initially G-PGMA displays a “few layers” structure, the introduction in the epoxy resin could lead to aggregates by reaction of the oxirane present in PGMA with the amine component of the resin. The enhanced magnetic properties of **MB014** can be explained by the increased amount of dopant presenting magnetic characteristics. Based on these explanations, a clear correlation about the determining factor for EMI shielding improvement cannot be made. Therefore, the next step consisted in the highlighting of aggregates formation by micro-CT analysis (Figure 9) for samples **MB013**, **MB014**, **MB015**, and **MB017**.

The largest aggregates can be observed for sample **MB013**, followed by **MB015** and **MB014**, while the most uniform sample appears to be **MB017**, all utilizing the “multilayer” type of functionalized graphene. Even if in the case of **MB013** large aggregates can be observed, the EMI shielding performance is similar to **MB017**. In the case of **MB017**, a very good dispersion of the doping agents can be observed (usually a determinant aspect for EMI), while in the case of **MB013** the good results good be attributed to an improvement of the electrical conductivity. Therefore, the electroconductive properties of the fillers counterbalance the lack of uniformity of the sample leading to a good EMI shielding efficiency. Thus, it can be concluded that G-HMDA presents an enhanced electrical conductivity compared with the unmodified graphene which is confirmed by similar examples in the literature data [47].

**Figure 10:**
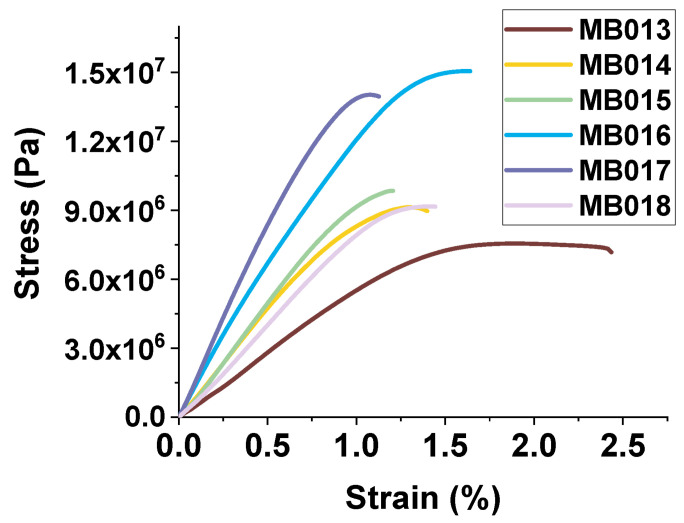
Comparative stress-strain plots of the thin films.

The analysis of the mechanical properties of the materials was also performed considering the application of the materials. Figure 10 illustrates a comparative stress-strain plot of epoxy thin films synthesized in this study. The analysis revealed that **MB013** is the most elastic sample, but it also presents the lowest mechanical resistance. This could be explained by an improved load transfer due to the presence of amino-functionalized graphene [48,49]. The addition of a supplementary amount of iron oxide to this formulation led to higher mechanical resistance (**MB014**) probably due to a reinforcing effect of the filler. On the other hand, a minor influence on the improvement of the mechanical performances of these thin films was observed when doubling the concentration of G-HDMA (sample **MB015**). In contrast with these first three materials, the samples containing G-PGMA (**MB016**) displayed superior mechanical performances, reaching a maximum value of approximately 1.5 × 10^7^ Pa tensile stress at 1.64% strain, probably due to the interactions established between the two polymers chain, the epoxy resin and the PGMA, grafted on the G surfaces, respectively. The samples containing neat G (**MB017**) resisted up to 1.4 × 10^7^ Pa tensile stress, the value measured at 1% strain. The mechanical behavior of **MB018** to tensile load is comparable to that of **MB014**. 

## 4. Conclusions

This study highlights the synthesis and characterization of epoxy-nanocomposites containing nanofillers of unmodified and functionalized graphene. The graphene modification involved both functional groups or small molecules and polymers for compatibilization with the epoxy resin components to provide electromagnetic interference (EMI) shielding effectiveness. The first part of the study was dedicated to the modification of graphene and its characterization by RAMAN and TGA. The results confirmed the obtaining of modified graphene with different thicknesses of the graphene sheets. Samples of graphene with different thicknesses (monolayer, few layers, and multilayer) were selected and further employed for epoxy coating formulation. The obtained nanocomposites were characterized in terms of EMI shielding effectiveness, SEM, micro-CT, magnetic properties, and stress-strain resistance. The interesting aspect is that the unmodified graphene and HMDA-modified graphene displayed the best EMI shielding properties, although the ununiformed characteristic of the nanocomposites was confirmed by the micro-CT. In addition, the epoxy nanocomposites based on HMDA-modified graphene displayed better flexibility with little difference in the EMI shielding effectiveness. The improved mechanical properties and EMI shielding characteristics make HMDA-graphene a good candidate for textile coatings with MA capacity.

## Figures and Tables

**Table 1:** EMI shielding samples codes and filler content.

Sample	Type of Graphene	Graphene Weight %	Iron Oxide Weight %
MB013	G-HMDA	5	2.5
MB014	G-HMDA	5	5
MB015	G-HMDA	10	5
MB016	G-PGMA	5	2.5
MB017	G	5	2.5
MB018	GO	5	2.5

**Table 2:** EMI shielding properties of different composite materials.

Matrix	Type of Filler Material	Filler wt%	Thickness of Sample	Frequency Range	Shielding Effectiveness (dB)	Ref.
Polyaniline (PANI)	Graphene and MWCNTs	5–10	2.5 mm	12.4–18 GHz	98	[39]
Wax	PANI/Fe3O4 and MWCNTs	20	4 mm	2–18 GHz	16	[40]
Mesocarbon microbead	MWCNTs	10–25	0.15–0.6	8.2–12.4 GHz	31–56	[41]
Epoxy resin	MWNCTs	4.2–20.4	0.35–1.75 mm		19–60	[42]
PANI:DBSA (dodecylbenzenesulfonic acid) mixed with divinyl benzene (DVB)	-		1.8	8.2–12.4 GHz	13	[43]
Polyvinyl butyral	Graphite/graphitized carbon black/Fe_2_O_3_	50/17/17	0.06 mm	0.3–4 GHz	9.1–17.8	[44]
PANI doped with poly (styrene sulfonic acid) (PSS) or HCl or HBr and Poly(3,4-ethylenedioxythiophene) (PEDOT) doped with PSS	Graphene	6–18	0.5 mm	3.4–6 GHz	60	[45]
Epoxy resin	Fe_3_O_4_ and functionalized Graphenes	2.5–10	0.1 mm	10.5–12.5 GhZ	4.5	This work

## Data Availability

Not applicable.

## References

[1] Rinaldi A., Proietti A., Tamburrano A., Sarto M.S. (2018). Graphene-Coated Honeycomb for Broadband Lightweight Absorbers. IEEE Trans. Electromagn. Compat..

[2] Singh S.K., Akhtar M.J., Kar K.K. (2019). Impact of Al2O3, TiO2, ZnO and BaTiO3 on the microwave absorption properties of exfoliated graphite/epoxy composites at X-band frequencies. Compos. Eng. Part B.

[3] Hisatake S., Nakajima H., Nguyen Pham H.H., Uchida H., Tojyo M., Oikawa Y., Miyaji K., Nagatsuma T. (2017). Mapping of electromagnetic waves generated by free-running self-oscillating devices. Sci. Rep..

[4] Teoh Y.J., Bruka M.A., Idris N.M., Ismail N.A., Muztaza N.M. (2018). Introduction of a Ground Penetrating Radar System for Subsurface Investigation in Balik Pulau, Penang Island. J. Phys. Conf. Ser..

[5] Pozar D.M. (2011). Microwave Engineering.

[6] Munir A. (2017). Microwave Radar Absorbing Properties of Multiwalled Carbon Nanotubes Polymer Composites: A Review. Adv. Polym. Technol..

[7] Qin M., Zhang L., Wu H. (2022). Dielectric Loss Mechanism in Electromagnetic Wave Absorbing Materials. Adv. Sci..

[8] Mirsaneh M., Furman E., Ryan J.V., Lanagan M.T., Pantano C.G. (2010). Frequency dependent electrical measurements of amorphous GeSbSe chalcogenide thin films. Appl. Phys. Lett..

[9] Wright P.V., Chambers B., Barnes A., Lees K., Despotakis A. (2000). Progress in smart microwave materials and structures. Smart Mater. Struct..

[10] Vendik O.G., Hollmann E.K., Kozyrev A.B., Prudan A.M. (1999). Ferroelectric Tuning of Planar and Bulk Microwave Devices. J. Supercond..

[11] Luo J., Zuo Y., Shen P., Yan Z., Zhang K. (2017). Excellent microwave absorption properties by tuned electromagnetic parameters in polyaniline-coated Ba0.9La0.1Fe11.9Ni0.1O19/reduced graphene oxide nanocomposites. RSC Adv..

[12] Prokopchuk A., Zozulia I., Didenko Y., Tatarchuk D., Heuer H., Poplavko Y. (2021). Dielectric Permittivity Model for Polymer–Filler Composite Materials by the Example of Ni- and Graphite-Filled Composites for High-Frequency Absorbing Coatings. Coatings.

[13] Jinxi H., Zhibo F. (1996). Input impedance of a tunable rectangular microstrip antenna. Wuhan Univ. J. Nat. Sci..

[14] Balci O., Polat E.O., Kakenov N., Kocabas C. (2015). Graphene-enabled electrically switchable radar-absorbing surfaces. Nat. Commun..

[15] Jayalakshmi C.G., Inamdar A., Anand A., Kandasubramanian B. (2019). Polymer matrix composites as broadband radar absorbing structures for stealth aircrafts. J. Appl. Polym. Sci..

[16] Sadiku E.R., Agboola O., Mochane M.J., Fasiku V.O., Owonubi S.J., Ibrahim I.D., Abbavaram B.R., Kupolati W.K., Jayaramudu T., Uwa C.A. (2021). The use of polymer nanocomposites in the aerospace and the military/defence industries. Research Anthology on Military and Defense Applications, Utilization, Education, and Ethics.

[17] Kumar S., Krishnan S., Samal S.K., Mohanty S., Nayak S.K. (2019). Polymer Nanocomposites Coating for Anticorrosion Application. Polymer Nanocomposites for Advanced Engineering and Military Applications.

[18] Stergiou C.A., Koledintseva M.Y., Rozanov K.N., Thakur V.K., Thakur M.K., Pappu A. (2017). 3-Hybrid polymer composites for electromagnetic absorption in electronic industry. Hybrid Polymer Composite Materials.

[19] Zhang K.-L., Zhang J.-Y., Hou Z.-L., Bi S., Zhao Q.-L. (2019). Multifunctional broadband microwave absorption of flexible graphene composites. Carbon.

[20] Balaji Ananth P., Abhiram N., Hari Krishna K., Nisha M.S. (2021). Synthesis of radar absorption material for stealth application. Mater. Today Proc..

[21] Zhang Y., Gu J. (2022). A Perspective for Developing Polymer-Based Electromagnetic Interference Shielding Composites. Nano-Micro Lett..

[22] Chen Y., Pang L., Li Y., Luo H., Duan G., Mei C., Xu W., Zhou W., Liu K., Jiang S. (2020). Ultra-thin and highly flexible cellulose nanofiber/silver nanowire conductive paper for effective electromagnetic interference shielding. Compos. Appl. Sci. Manuf..

[23] Xia X., Li Y., Long L., Xiao P., Luo H., Pang L., Xiao X., Zhou W. (2020). Modeling for the electromagnetic properties and EMI shielding of Cf/mullite composites in the gigahertz range. J. Eur. Ceram. Soc..

[24] Shi M., Feng C.-P., Li J., Guo S.-Y. (2022). Machine learning to optimize nanocomposite materials for electromagnetic interference shielding. Compos. Sci. Technol..

[25] Silva V.A., Folgueras L.d.C., Cândido G.M., Paula A.L.d., Rezende M.C., Costa M.L. (2013). Nanostructured composites based on carbon nanotubes and epoxy resin for use as radar absorbing materials. Mater. Res..

[26] Shen B., Zhai W., Zheng W. (2014). Ultrathin Flexible Graphene Film: An Excellent Thermal Conducting Material with Efficient EMI Shielding. Adv. Funct. Mater..

[27] Batrakov K., Kuzhir P., Maksimenko S., Paddubskaya A., Voronovich S., Lambin P., Kaplas T., Svirko Y. (2014). Flexible transparent graphene/polymer multilayers for efficient electromagnetic field absorption. Sci. Rep..

[28] Chen Z., Xu C., Ma C., Ren W., Cheng H.-M. (2013). Lightweight and Flexible Graphene Foam Composites for High-Performance Electromagnetic Interference Shielding. Adv. Mater..

[29] Li J.-S., Huang H., Zhou Y.-J., Zhang C.-Y., Li Z.-T. (2017). Research progress of graphene-based microwave absorbing materials in the last decade. J. Mater. Res..

[30] Muhammed Shameem M., Sasikanth S.M., Annamalai R., Ganapathi Raman R. (2021). A brief review on polymer nanocomposites and its applications. Mater. Today Proc..

[31] Shukla P., Saxena P. (2021). Polymer Nanocomposites in Sensor Applications: A Review on Present Trends and Future Scope. Chin. J. Polym. Sci..

[32] Hiremath A., Murthy A.A., Thipperudrappa S., NK B. (2021). Nanoparticles Filled Polymer Nanocomposites: A Technological Review. Cogent Eng..

[33] Mouritz A.P. (2012). 13-Polymers for aerospace structures. Introduction to Aerospace Materials.

[34] Peng C., Zhang X. (2021). Chemical Functionalization of Graphene Nanoplatelets with Hydroxyl, Amino, and Carboxylic Terminal Groups. Chemistry.

[35] Xue H., Lu Y., Geng H., Dong B., Wu S., Fan Q., Zhang Z., Li X., Zhou X., Wang J. (2019). Hydroxyl Groups on the Graphene Surfaces Facilitate Ice Nucleation. J. Phys. Chem. Lett..

[36] Shayimova J., Amirov R.R., Iakunkov A., Talyzin A., Dimiev A.M. (2021). Carboxyl groups do not play the major role in binding metal cations by graphene oxide. Phys. Chem. Chem. Phys..

[37] Liang S., Gao P., Wang A., Zeng C., Bao G., Tian D. (2022). Insights into the influence of functional groups on the properties of graphene from first-principles calculations. J. Phys. Org. Chem..

[38] Chen J., Zhang Y., Zhang M., Yao B., Li Y., Huang L., Li C., Shi G. (2016). Water-enhanced oxidation of graphite to graphene oxide with controlled species of oxygenated groups. Chem. Sci..

[39] Gupta T.K., Singh B.P., Mathur R.B., Dhakate S.R. (2014). Multi-walled carbon nanotube–graphene–polyaniline multiphase nanocomposite with superior electromagnetic shielding effectiveness. Nanoscale.

[40] Cao M.-S., Yang J., Song W.-L., Zhang D.-Q., Wen B., Jin H.-B., Hou Z.-L., Yuan J. (2012). Ferroferric Oxide/Multiwalled Carbon Nanotube vs Polyaniline/Ferroferric Oxide/Multiwalled Carbon Nanotube Multiheterostructures for Highly Effective Microwave Absorption. ACS Appl. Mater. Interfaces.

[41] Chaudhary A., Kumari S., Kumar R., Teotia S., Singh B.P., Singh A.P., Dhawan S.K., Dhakate S.R. (2016). Lightweight and Easily Foldable MCMB-MWCNTs Composite Paper with Exceptional Electromagnetic Interference Shielding. ACS Appl. Mater. Interfaces.

[42] Singh B.P., Prasanta V., Choudhary V., Saini P., Pande S., Singh V.N., Mathur R.B. (2013). Enhanced microwave shielding and mechanical properties of high loading MWCNT–epoxy composites. J. Nanopart. Res..

[43] Das S., Sharma S., Yokozeki T., Dhakate S. (2021). Conductive layer-based multifunctional structural composites for electromagnetic interference shielding. Compos. Struct..

[44] Khomenko V., Butenko O., Chernysh O., Barsukov V., Suchea M.P., Koudoumas E. (2022). Electromagnetic Shielding of Composite Films Based on Graphite, Graphitized Carbon Black and Iron-Oxide. Coatings.

[45] Tudose I.V., Mouratis K., Ionescu O.N., Romanitan C., Pachiu C., Popescu M., Khomenko V., Butenko O., Chernysh O., Kenanakis G. (2022). Novel Water-Based Paints for Composite Materials Used in Electromagnetic Shielding Applications. Nanomaterials.

[46] Singh K., Ohlan A., Dhawan S. (2012). Polymer-graphene nanocomposites: Preparation, characterization, properties, and applications. Nanocompos.-New Trends Dev..

[47] Rabchinskii M.K., Ryzhkov S.A., Kirilenko D.A., Ulin N.V., Baidakova M.V., Shnitov V.V., Pavlov S.I., Chumakov R.G., Stolyarova D.Y., Besedina N.A. (2020). From graphene oxide towards aminated graphene: Facile synthesis, its structure and electronic properties. Sci. Rep..

[48] Chatterjee S., Wang J.W., Kuo W.S., Tai N.H., Salzmann C., Li W.L., Hollertz R., Nüesch F.A., Chu B.T.T. (2012). Mechanical reinforcement and thermal conductivity in expanded graphene nanoplatelets reinforced epoxy composites. Chem. Phys. Lett..

[49] Wazalwar R., Sahu M., Raichur A.M. (2021). Mechanical properties of aerospace epoxy composites reinforced with 2D nano-fillers: Current status and road to industrialization. Nanoscale Adv..

